# A six-month low-carbohydrate diet high in fat does not adversely affect endothelial function or markers of low-grade inflammation in patients with type 2 diabetes: an open-label randomized controlled trial

**DOI:** 10.1186/s12933-023-01956-8

**Published:** 2023-08-17

**Authors:** Eva M. Gram-Kampmann, Thomas B. Olesen, Camilla D. Hansen, Mie B. Hugger, Jane M. Jensen, Aase Handberg, Henning Beck-Nielsen, Aleksander Krag, Michael H. Olsen, Kurt Højlund

**Affiliations:** 1 Steno Diabetes Center Odense, Odense University Hospital, Kløvervænget 10, Entrance 112, 5000 Odense, Denmark; 2 Department of Clinical Research, University of Southern Denmark, Odense, Denmark; 3 Open Patient Data Explorative Network, Odense University Hospital, Odense, Denmark; 4 Department of Gastroenterology and Hepatology, Odense University Hospital, Odense, Denmark; 5 Department of Clinical Biochemistry, Aalborg University Hospital, Ålborg, Denmark; 6 Department of Clinical Medicine, Aalborg University, Ålborg, Denmark; 7 Department of Internal Medicine 1, Holbæk Hospital, and Steno Diabetes Center Zealand, Holbæk, Denmark; 8 Department of Regional Health Research, University of Southern Denmark, Odense, Denmark

**Keywords:** Low-carbohydrate diet, Type 2 diabetes, Endothelial function, Flow-mediated vasodilation, Low-grade inflammation, Cardiovascular risk

## Abstract

**Background:**

While a low-carbohydrate diet (LCD) reduces HbA1c in patients with type 2 diabetes (T2D), the associated high intake of fat may adversely affect cardiovascular risk factors. To address this, we examined the effect of a non-calorie-restricted LCD high in fat on endothelial function and markers of low-grade inflammation in T2D over 6 months.

**Methods:**

In an open-label randomized controlled trial, 71 patients with T2D were randomized 2:1 to either a LCD (< 20 E% carbohydrates, 50–60 E% fat) or a control diet (50–60 E% carbohydrates, 20–30 E% fat) for six months. Flow-mediated vasodilation (FMD) and nitroglycerine-induced vasodilation (NID) were assessed by ultrasound in the brachial artery together with plasma interleukin-6 (IL-6) and serum high-sensitivity C-reactive protein (hsCRP) in the participants at baseline (n = 70) and after six months (n = 64).

**Results:**

The FMD and NID were unaltered in both groups after six months, and there were no between-group differences in change of either FMD (p = 0.34) or NID (p = 0.53) in response to the interventions. The circulating hsCRP and IL-6 levels decreased only in response to LCD (both p < 0.05). However, comparing changes over time with the control diet, the LCD did not reduce either IL-6 (p = 0.25) or hsCRP (p = 0.07) levels. The lack of changes in FMD and NID in response to LCD persisted after adjustment for cardiovascular risk factors.

**Conclusion:**

A LCD high in fat for six months does not adversely affect endothelial function or selected markers of low-grade inflammation, which suggests that this nutritional approach does not increase the risk of cardiovascular disease.

*Trial registration* ClinicalTrials.gov (NCT03068078).

**Supplementary Information:**

The online version contains supplementary material available at 10.1186/s12933-023-01956-8.

## Introduction

One of the major causes of mortality in type 2 diabetes (T2D) is cardiovascular disease (CVD) [1], which contributes to a reduced life span [2]. A number of factors contribute to an increased risk of CVD in patients with T2D such as hypertension, dyslipidemia, poor glycemic control, abdominal obesity and insulin resistance [3]. These factors are associated with chronic low-grade inflammation as indicated by increased circulating levels of pro-inflammatory markers such as high-sensitivity C-reactive protein (hsCRP) and interleukin-6 (IL-6) [4]. The endothelium is highly susceptible to low-grade inflammation, and endothelial dysfunction is one of the earliest signs of progressing atherosclerosis [5]. In endothelial dysfunction, the normal regulation of the vessel tone, inflammatory processes and anticoagulation to maintain vascular homeostasis are disrupted [6, 7], with impaired ability to react to physiological stimuli [8]. Flow-mediated vasodilation (FMD) in the brachial artery, measured as the percentage change in artery diameter after reactive hyperemia, is significantly lower in hypertensive patients with T2D compared with hypertensive persons without T2D [9]. Reduced FMD is recognized as an independent risk factor for future development of CVD such as cardiovascular death, myocardial infarction, need of revascularization of coronary arteries, and stroke [5, 10, 11].

T2D management includes lifestyle changes involving both diet and physical activity and is important to improve glycemic control and decrease risk of CVD [3]. Low-carbohydrate diets have beneficial effects on HbA1c compared to control diets over 6 months [12], but the increase in dietary fat, in particular saturated fat, have raised concerns about an increased risk of developing CVD [13]. Smaller cross-sectional studies have shown that a higher habitual intake of dietary fat [14], and saturated fat [15] is associated with impaired endothelial function, whereas the effect of substituting meals high in saturated fat with meals high in monounsaturated fatty acids (MUFA) on vascular function remains to be established [16]. Moreover, the reported effects of carbohydrate-restricted diets high in fat on endothelium-dependent vasodilation are primarily in non-diabetic individuals and inconsistent. Thus, in healthy obese individuals, low carbohydrate diets did not affect FMD over six weeks [17], 12 weeks [18] nor 12 months [19], regardless of whether it included additional approaches such as a reduced caloric intake or increased exercise or not. In contrast, a meta-analysis found decreased FMD in response to carbohydrate-restricted diets (≤ 45 E% carbohydrate) [20], while another report pointed to improved vasoreactivity after a very low-carbohydrate diet (VLCD) for 6 weeks compared to a low-fat diet [21]. In individuals with T2D, the effect of carbohydrate-restricted diets high in fat on measures of endothelial function is not very well examined. Wycherley et al.reported no effect on brachial FMD in patients with T2D randomized to a VLCD (14 E% carbohydrates) compared with a high-carbohydrate diet low in fat after 12 months [22], and with no effect on endothelial function in either groups after 24 months [23]. However, in both groups, the diets were energy-restricted (~ 30%) and combined with a supervised exercise program leading to significant but similar reductions in weight and HbA1c, which may have obscured the true effect of the diets on FMD [22–24]. Interestingly, Barbosa-Yañez et al. [25] found that the brachial FMD increased more than 50% after three weeks of a hypocaloric low fat diet compared with no change in response to a hypocaloric VLCD (5–10 E% carbohydrates) in patients with T2D suggesting a potential harmful effect of carbohydrate-restriction on endothelial function.

Along with endothelial dysfunction, circulating markers of low-grade inflammation such as hsCRP and IL-6 are often elevated in obesity and T2D and associated with a higher risk of cardiovascular events [26–28]. So far, studies comparing the effect of carbohydrate restriction with low fat diets on circulating hsCRP and IL-6 levels in patients T2D have been scarce and inconclusive [29, 30]. In particular, the effect of restricting carbohydrate intake to 10–25 E% (defined as a low-carbohydrate diet [12, 31], LCD) on these pro-inflammatory markers in patients with T2D remains to be established.

Previously, we reported that in patients with T2D a LCD high in fat for 6 months reduced HbA1c, weight and abdominal adiposity compared to a control diet, while blood pressure and lipid levels were unaffected [32]. In the present study, we aimed to investigate whether a LCD high in fat for 6 months adversely affects CVD risk factors such as endothelial function evaluated as FMD and nitroglycerine-dependent dilation (NID) and markers of chronic low-grade inflammation compared to a control diet in patients with T2D, who were instructed to maintain their energy intake and physical activity levels.

## Methods

### Study design and participants

As previously reported [32] the study was an out-patient, open-labelled (unblinded), randomized controlled trial (RCT) of the effects of a LCD high in fat compared to a control diet low in fat for 6 months in patients with T2D. The study was conducted from November 2016 to May 2019 at Odense University Hospital, Odense, Denmark. Participants with established T2D were recruited through mainly public advertisement and social media, in addition to invitations to patients, who had attended previous studies. Results from this study including changes in glycaemic control, measures of body composition, blood lipids and blood pressure were recently reported [32]. In the present study, we report the prespecified secondary outcome endothelial function as well as markers of low-grade inflammation.

Briefly described, the inclusion criteria included an established diagnosis of T2D [33], age older than 18 years, an HbA1c of more than 48 mmol/mol, a diabetes duration of six months to five years, but up to 10 years if treated with ≤ 2 non-insulin antidiabetic drugs, and stable glucose lowering therapy > 3 months prior to inclusion. To prevent changes in cholesterol lowering treatment during the study, the inclusion criteria included a LDL cholesterol < 2.5 mmol/l and a total cholesterol < 4.5 mmol/l. However, if the patients could not tolerate statin and/or refused treatment, a higher LDL-cholesterol was accepted. Exclusion criteria were significant co-morbidities or significant diabetic comorbidities that could risk safety during diet change or affect diet compliance. Other exclusion criteria were continuous use of steatosis-inducing drugs or glucocorticoids, following a carbohydrate-restricted diet prior to inclusion, excessive weight loss before enrollment (defined as > 10 kilos over 3 months) or pregnancy/planned pregnancy.

Out of 345 persons eligible for screening [32], 73 were enrolled and randomized 2:1, stratified on sex and number of antidiabetic drugs (0–1 and ≥ 2), to either the LCD intervention or a control diet. Two sets of family members were randomized as one unit each, and two participants withdrew consent before commencing to the study, leaving 49 participants for inclusion in the LCD group and 22 in the control group at baseline. After 6 months of the study, five in the LCD group and two in the control group had dropped out [32].

Informed consent was obtained from all individuals before participation. The study was approved by the Regional Committees on Health Research Ethics for Southern Denmark and was performed in accordance with the Declaration of Helsinki Declaration II. The RCT was registered at ClinicalTrials.gov (NCT03068078).

### Diet intervention and physical activity

This was a free-living study and no food was provided to the participants. The LCD group was instructed to follow a diet consisting of a maximum of 20 E% carbohydrates, 50–60 E% fats 25–30 E% protein with a recommendation of a high intake monounsaturated fatty acids (MUFAs) and as low intake of saturated fatty acids (SFA) as possible [32]. The control diet group were instructed to follow a diet according to the current official Danish dietary guidelines with a recommended intake of 50–60 E% carbohydrates, 20–30 E% fat, where < 10 E% should be from saturated fat, and 20–25 E% protein [34].

All participants were individually introduced to the diet by a licensed clinical dietitian, had opportunity for on-demand visits and could attend group-specific discussion meetings supervised by the dietitian. The dietitian contacted every participant per telephone one week after starting the new diet and hereafter every month. A five-days startup menu plan based on pre-study calorie intake was provided as well as weekly newsletters to all participants. Participants had access to a recipe-database that was continuously updated and were instructed to register food intake in MadLog (MadLog ApS, Kolding, Denmark). Based on the estimated energy requirement at baseline, participants were guided to maintain their calorie-intake during the entire intervention. Furthermore, the participants were instructed maintain their usual physical activity level throughout the study period. As reported [32], this was examined by accelerometer-based assessment of physical activity for seven days at baseline and after 6 months. For further details regarding the diet and accelerometry please refer to our previous report [32].

### Assessment of flow-mediated vasodilation (FMD and nitroglycerine induced dilation (NID)

FMD and NID in the brachial artery were assessed by a single investigator at baseline and after 6 months of diet change using a Phillips iE33 ultrasound machine with a L15-7io linear array transducer and automated settings for FMD/NID. The participant’s right arm was examined in the morning after an overnight fast, minimum 8 h. The participants were instructed to discontinue antihypertensive medication, vitamins and sildenafil three days before the examination and to refrain from strenuous exercise, tea and juice for 48 h and coffee, alcohol and nicotine for 12 h prior to the examination. Any ongoing cholesterol-lowering treatment continued but was not taken on the day of examination. One participant was examined at noon both times.

Following a 15 min rest in supine position, blood pressure was measured in the left arm to ensure cuff inflation minimum 20 mmHg above systolic blood pressure (minimum 200 mmHg). A rapid inflation/deflation cuff was applied with upper crease in the cubital fossa on the right forearm (Hokanson E20, Bellevue USA), where after a suitable segment of the brachial artery proximal of the cubital fossa was identified. Anatomical markers and cuff pressure were noted for follow-up examinations. The resting brachial artery diameter (RD_FDM_) was recorded at least three times for 60 s, lifting the transducer between each recording. The cuff was then inflated for five minutes and recordings were resumed five seconds before cuff deflation and continued for five minutes to determine FMD. The resting brachial artery (RD_NID_) was assessed after another 15-min rest with another three recordings after which one spray of 400 µg of sublingual glyceryl trinitrate was administered. After that the recordings continued for 9.4 min (6 × 94 s) to determine NID.

Sequences were exported as AVI-files or DICOM for off-line analyses. The same trained person who executed the FMD measurements also analyzed offline the individual sequences blinded for patient ID and clinical data using a semi-automated, commercial software (Brachial Analyzer, Medical Imaging Application, version 6.9.1, Coralville, Iowa, USA) [35]. Auto-gated, end-diastolic diameters were used. To examine intra-observer reliability for FMD and NID, a random sample of twelve volunteers were scanned on two consecutive days, assessing FMD. The resulting intra-observer reliability coefficient was 0.968, which is similar to that reported in similar studies [36].

The following variables were estimated: The resting diameters (RD_FMD_, RD_NID_) were calculated by using the mean of the 60-s resting end-diastolic diameter measurements. The maximal flow-mediated vasodilation (FMD_max_) was calculated as the mean of minimum five consecutive auto-gated diameters after reaching peak dilation, which was obtained through visual inspection of the end-diastolic diameters. The maximal nitroglycerine-induced dilation (NID_max_) was estimated as the mean of minimum 60 s measurements after the individual peak dilation was reached. FMD and NID were calculated and reported as the percentage change (%) compared to the resting diameter. The ratio between FMD and NID (FMD/NID) was calculated as an estimate of endothelial-dependent vasodilatory function adjusted for endothelium-independent vasodilatory capacity. The cut-off values for FMD and NID used to distinguish those without an increased risk of CVD from those with an increased risk, were FMD < 7.1% and NID < 15.6%, respectively [37].

Out of the 71 FMD and NID sequences obtained at baseline, the FMD and NID in one person from the control group could not be analyzed due to low image quality and the NID from one person in the LCD group was missing due to damaged files (LCD), leaving a total of 70 FMD and 69 NID sequences for analysis at baseline. Given the seven drop-outs (see above), 64 FMD- and NID sequences were available for analysis at the 6 month follow-up. None of the participants FMD- or NID data were excluded due to outliers.

### Other outcome measures

All participants attended three visits during the study (baseline, 3 months and 6 months) with collection of fasting blood samples for measuring lipids, insulin, HbA1c, fasting plasma glucose and blood-ketones as reported [32]. Anthropometric measurements were assessed at all three visits. Participants were scanned with a dual-energy x-ray absorptiometry (DXA) scan (Hologic, Marlborough, MA) at baseline and after 6 months.

Serum hsCRP was measured in duplicates by an in-house ELISA using commercially available monoclonal antibodies and reagents (Biotechne, R&D Systems, MN, USA) according to the manufacturers instructions. The limit of detection was 0.05 µg/L and the intra- and inter-assay CVs were below 15%. Circulating IL-6 was measured in singlets on fasting EDTA plasma by the human high-sensitive IL-6 ELISA assay essentially as described (R&D Systems, Abingdon, UK). Mean CV% between runs was 6.9% (EDTA plasma pool, level 8.1 pg/ml). CV% of assay controls were 20% (level 0.5 pg/ml), 12.6%, (level 3.2 pg/ml) and 18.5% (level 5.9 pg/ml).

### Statistical analysis

Statistical analysis was done with STATA for Windows (STATA 16.0, StataCorp LLC, Texas, USA). The vascular function was one of the pre-specified secondary outcomes in this study. The estimation of the sample size was based on the primary outcome, HbA1c, and this showed that 36 in the LCD group and 18 in the control group were sufficient to obtain a power of 80% as reported [32].

All residuals were tested for normal distribution, and if the residuals did not meet criteria of normal distribution, the dependent variables were log-transformed prior to the statistical analyses. For analyses of changes over time within and between groups, a mixed model with randomization- and time interaction was applied. The mean difference in change (MDIC) between groups from baseline to 6 month is reported as the effect of LCD versus control diet. The relationship between FMD and NID and several measured CVD risk factors including circulating IL-6 and hsCRP levels at baseline was examined using univariate linear regression or Spearman’s rank correlation coefficient if residuals were not normally distributed. Estimates of MDIC and linear regressions are reported as coefficients ± SE. All other data are reported as mean ± SD. Statistical significance was assumed at p < 0.05.

## Results

### Baseline characteristics

Among study participants with valid FMD measurements at baseline (n = 70), the two groups were comparable with respect to age, duration of diabetes, HbA1c, gender distribution, smoking status, lipid levels and BMI (Table 1 and Additional file 1: Table S1). The systolic blood pressure was higher in the control group at baseline. As reported [32], there were no differences in the types or number of glucose- or blood pressure lowering drugs between the groups.

**Table 1:** Baseline characteristics

	LCD (n = 49)	Control (n = 21)
Age (years)	55.2 ± 6.2	57.1 ± 12.9
Duration of type 2 diabetes (years)	5.2 ± 3.2	5.0 ± 2.4
Female sex	26 (53)	12 (57)
Smoking status		
Never smoked	24 (49)	10 (48)
Active smoker	2 (4)	1 (5)
Exsmoker	23 (47)	10 (48)
Smoking pack years (years)	21.4 ± 15.7	14.0 ± 17.1
Body Mass Index (kg/m^2^)	32.5 ± 6.0	35.5 ± 6.8
Heart rate (bpm)	77 ± 11.8	83 ± 16.8
Hypertension treatment	32 (65)	15 (71)
No. of blood pressure lowering agents		
0	17 (35)	6 (29)
1	14 (29)	7 (33)
2	10 (20)	5 (24)
3	5 (10)	1 (5)
4	2 (4)	2 (10)
5	1 (2)	0 (0)
No. of glucose-lowering agents		
0	5 (10)	3 (14)
1	23 (47)	11 (52)
2	16 (33)	4 (19)
> 3	5 (10)	3 (14)
Cholesterol-lowering treatment	27 (55)	11 (52)

Data are means ± SD or number (%)

### Changes in clinical parameters

As reported previously [32], the LCD resulted in a reduction of HbA1c, weight, BMI, total fat mass and waist circumference after 6 months compared to the control diet, whereas no changes in blood lipids, or blood pressure (Additional file 1: Table S1). As also reported before [32], there was no change in the physical activity level in response to LCD (data not shown). After 6 months, 35 of 44 (80%) in the LCD groups and 8 of 20 (40%) in the control group had HbA1c ≤ 48 mmol/mol (p = 0.002). The LCD group reduced their self-reported carbohydrate intake to 13.4 E% while fat intake was increased to 63.2 E% compared to an intake of 48.4 E% carbohydrate and 28.3 E% of fat in the control group after 6 months [32]. The increased intake of fat in the LCD group was explained by an increased intake of not only SFA, but also MUFA and polyunsaturated fatty acids (PUFA) compared with the control diet (Additional file 1: Table S1) [32]. At the end of the study, the self-reported intake of SFA in both E% and g/day was 2.6 fold higher in the LCD group compared with the control group [32].

### Measures of endothelial function

**Fig. 1:**
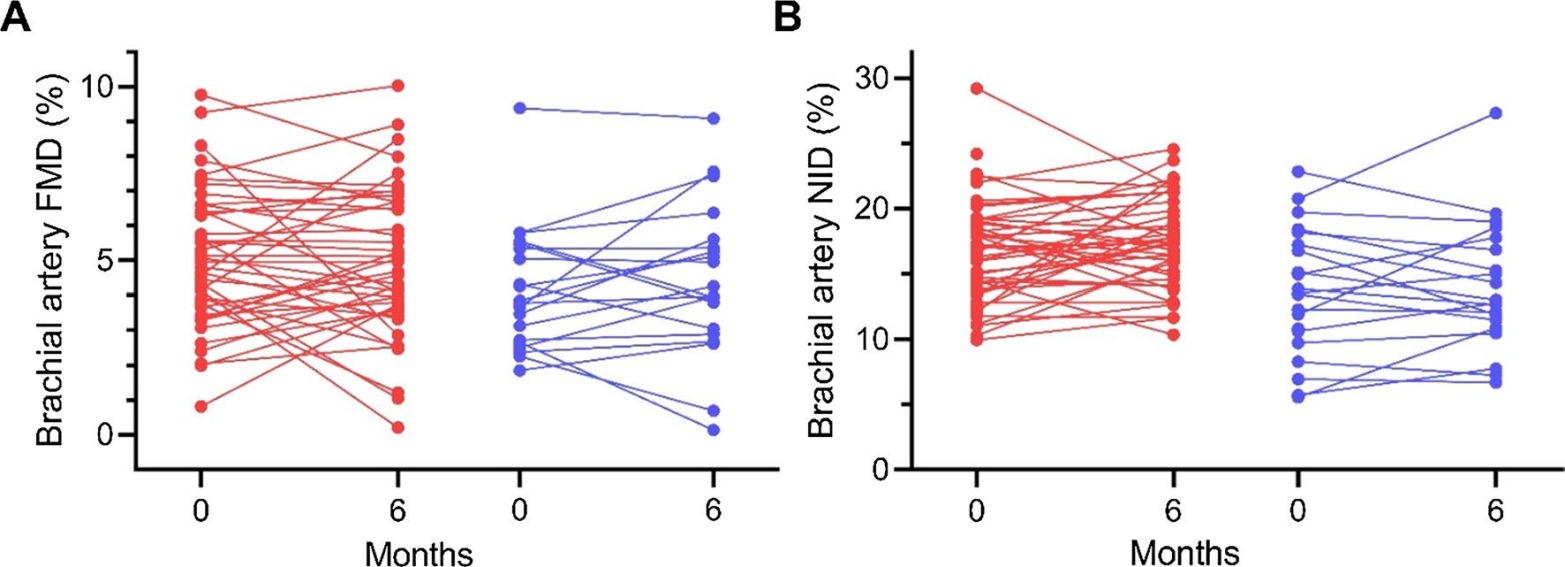
Individual changes in **A** flow-mediated vasodilation (FMD) and **B** nitroglycerine-induced dilation (NID) from baseline to 6 months in patients with type 2 diabetes randomized to either a LCD (red circles/lines) or a control diet (blue circles/lines)

At baseline, 41 of 49 (84%) in the LCD group and 20 of 21 (95%) in the control group had FMD < 7.1% (p = 0.42), and 20 of 48 (42%) in the LCD group and 14 of 21 (67%) in the control group had NID < 15.6% (p = 0.06). These proportions did not change significantly after 6 months (data not shown). In the LCD group, the resting diameters (RD_FMD_ and RD_NID_) and maximal dilations (FMD_max_ and NID_max_) decreased, whereas FMD and NID were unaltered after 6 months on the diet. In the control group, no changes were observed (Table 2). Moreover, the FMD/NID ratios did not change in either of the groups after 6 month. Importantly, there were no effects of LCD on the resting diameters nor maximal dilations (all p > 0.46), nor on FMD (MDIC: -0.44 ± 0.47%, p = 0.34), NID (MDIC: + 0.59 ± 0.93%, p = 0.53) nor FMD/NID (MDIC: -0.02 ± 0.04, p = 0.52) compared with the control diet (Fig. 1 and Table 2).

**Table 2:** Baseline and effects of 6 months the LCD and control diet on endothelial function

	Baseline		6 months			
	LCD (n = 49)	Control (n = 21)	LCD (n = 44)	Control (n = 20)	MDIC	p-value
RD_FMD_ (mm)	4.28 ± 0.75	4.37 ± 0.66	4.17 ± 0.73*	4.35 ± 0.67	+ 0.00 ± 0.04	0.93
FMD max (mm)	4.50 ± 0.76	4.55 ± 0.68	4.38 ± 0.76**	4.54 ± 0.67	− 0.01 ± 0.04	0.85
FMD (%)	5.19 ± 1.97	4.17 ± 1.79	5.00 ± 2.14	4.52 ± 2.23	− 0.44 ± 0.47	0.34
RD_NID_ (mm)	4.29 ± 0.74	4.39 ± 0.65	4.14 ± 0.74**	4.37 ± 0.69	− 0.04 ± 0.05	0.46
NID max (mm)	5.00 ± 0.83	4.98 ± 0.71	4.86 ± 0.83*	4.97 ± 0.73	− 0.02 ± 0.06	0.75
NID (%)	16.66 ± 3.90	13.64 ± 4.91	17.47 ± 3.43	14.03 ± 4.92	+ 0.59 ± 0.93	0.53
FMD%/NID%	0.32 ± 0.12	0.33 ± 0.15	0.29 ± 0.12	0.33 ± 0.15	− 0.02 ± 0.04	0.52

MDIC are reported as β-coefficient ± SE

Other data as mean ± SD

FMD, flow-mediated vasodilation; LCD, low carbohydrate diet; MDIC, mean difference in change; NID, nitroglycerin-induced vasodilation; RD, resting diameter

*p < 0.05 or **p < 0.01 vs baseline

### Measures of low-grade inflammation

**Fig. 2:**
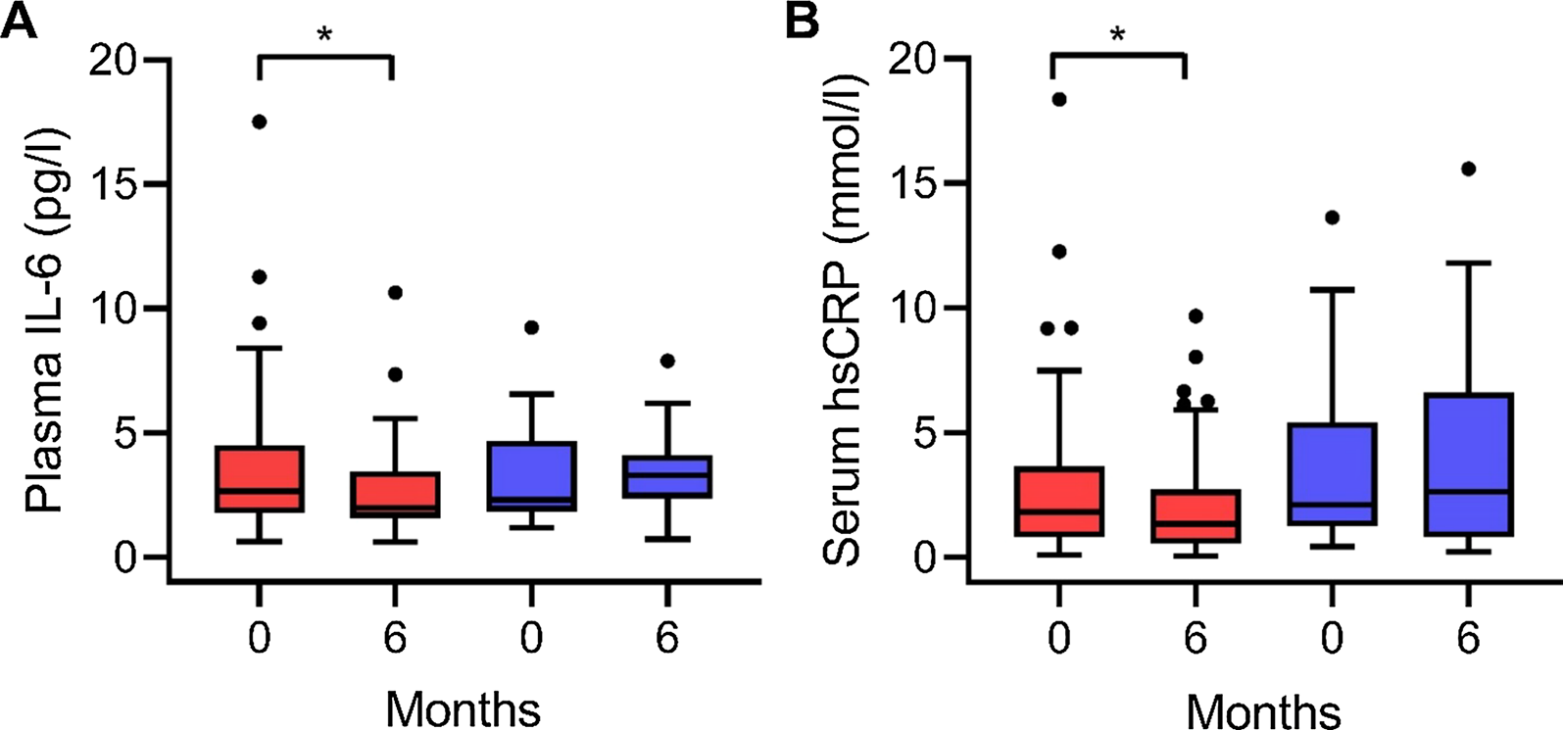
Changes in **A** plasma IL-6 and **B** serum hsCRP from baseline to 6 months in patients with type 2 diabetes randomized to either a LCD (red boxplots) or a control diet (blue bloxplots). *p < 0.05, within group change

In the LCD group, both hsCRP (p = 0.004) and IL-6 (p = 0.013) decreased after 6 months on the diet, whereas no significant changes were seen in the control group (Fig. 2). However, when comparing changes over time, the LCD did not significantly reduce neither IL-6 (MDIC: p = 0.247) nor hsCRP (MDIC: p = 0.065) compared with the control diet.

Using Spearman’s rank correlation coefficient in the total cohort (n = 70) at baseline, we found that plasma Il-6 levels correlated positively with BMI (p < 0.001), and abdominal (p = 0.004) and total body fat percentage (p = 0.011), but not with the other CVD risk factors listed in Table 3. The serum levels of hsCRP correlated positively with female sex (p = 0.036), triglycerides (p = 0.024), BMI (p < 0.001), and abdominal (p < 0.001) and total body fat percentage (p < 0.001).

**Table 3:** Association of endothelial function with cardiovascular risk factors

	FMD (%)	NID (%)
	Coefficients ± SE	Coefficients ± SE
Sex (female)	0.42 ± 0.47	− 0.35 ± 1.07
Type 2 diabetes duration (years)	− 0.04 ± 0.08	− 0.09 ± 0.18
Age (years)	− 0.08 ± 0.03**	− 0.13 ± 0.06*
Smoking status		
Non-smoker	(Base)	(Base)
Active smoker	− 0.16 ± 1.20	2.79 ± 2.61
Previous smoker	− 0.03 ± 0.49	2.15 ± 1.07*
Smoking pack years (years)	− 0.01 ± 0.02	− 0.05 ± 0.04
BMI (kg/m^2^)	− 0.01 ± 0.04	− 0.07 ± 0.08
Systolic blood pressure (mmHg)	− 0.05 ± 0.02**	− 0.06 ± 0.04
Diastolic blood pressure (mmHg)	− 0.04 ± 0.03	− 0.04 ± 0.06
LDL-cholesterol (mmol/l)	− 0.11 ± 0.31	0.48 ± 0.70
Triglycerides (mmol/l)	0.08 ± 0.20	0.39 ± 0.44
HbA1c (mmol/mol)	− 0.01 ± 0.03	− 0.00 ± 0.06
Resting diameter (mm)	− 0.91 ± 0.31**	− 1.89 ± 0.72*
Body fat (%)	0.01 ± 0.03	− 0.03 ± 0.07
Abdominal fat (%)	0.01 ± 0.03	− 0.04 ± 0.07
hsCRP (mmol/l)	0.01 ± 0.07	− 0.06 ± 0.15
IL-6 (pg/l)	0.11 ± 0.09	− 0.09 ± 0.20

hsCRP, high sensitivity CRP

*p < 0.05, **p < 0.01

### Adjusting for covariates correlated to FMD or NID

To adjust the analysis for between-group differences in CVD risk factors at baseline, we evaluated the relationship between FMD and NID and all the CVD risk factors listed in Table 3. This univariate linear regression analysis showed that FMD correlated significantly with age, systolic blood pressure, and RD_FMD_ at baseline, whereas NID correlated significantly with age and RD_NID_.

When adjusting our analysis for these significant covariates, there was, however, still no effect of LCD for 6 months on FMD (adjusted MDIC: − 0.44 ± 0.45%, p = 0.335) or NID (adjusted MDIC: + 0.56 ± 0.92%, p = 0.543).

### Description of changes in cholesterol and blood pressure lowering medication

There were no changes in cholesterol-lowering treatment during the study, except for two LCD participants who reported sporadic use of statins. In the LCD-group, one patient treated with both an angiotensin-converting enzyme (ACE) inhibitor and an angiotensin II receptor blocker (ARB) had the ACE inhibitor discontinued. Another patient in the LCD group discontinued thiazide treatment and was reduced in beta-blocker treatment. In two other patients in the LCD group, the dose of ARB treatment dose reduced (25 mg and 50 mg, respectively) due to orthostatism. In one patient in the control group, the dosage of ACE inhibitor combined with hydrochlorthiazide was increased.

## Discussion

In this second study from an open-label RCT [32], we report the effect of a 6-months non-calorie-restricted LCD on measures of endothelial function in the brachial artery and selected markers of low-grade inflammation compared to a control diet in patients with T2D. The LCD did not result in changes in either endothelium-dependent or endothelium-independent arterial dilatation assessed as brachial artery FMD and NID, respectively, compared to the control diet in patients with T2D. Although circulating IL-6 and hsCRP levels decreased only the LCD group, there were no significant between-group differences in the change of IL-6 or hsCRP levels over time. These findings together with the previously published lack of changes in blood lipids and blood pressure in this study support that a non-calorie-restricted LCD without changes in physical activity does not increase the risk of CVD despite a high intake of fat, including both SFA, MUFA and PUFA. However, longer studies are needed to confirm these findings beyond 6 months.

Population-based studies have provided evidence that correction of a suboptimal diet may be a powerful approach to reduce the risk of CVD [38]. Our findings extend results from the reports by Wycherley et al. [22] and Tay et al. [23], who found no between-group differences in change of brachial FMD when comparing a VLCD low in saturated fat to a high-carbohydrate diet low in fat, both combined with calorie-restriction and a supervised exercise program in patients with T2D. In the study by Wycherley et al. [22], the authors speculated that this might be due to a significant weight loss observed in both groups during the study, in addition to a decreased intake of saturated fat while maintaining intake of dietary fibers. The prescribed exercise program in both groups may also have affected the outcome in these studies [22, 23], as exercise training has been demonstrated to improve endothelial function [39]. In the present study, the participants in both groups were instructed to maintain their level of physical activity and intake of calories according to their baseline energy requirement, and the LCD group increased their intake of saturated fat and lost weight compared to the control group. These findings suggest that the lack of changes in FMD reported in the previous reports [22, 23] may not be explained by a calorie restriction-induced weight loss or exercise training, and that neither an increase nor a decrease in saturated fat in these diets affect endothelial function. In support, a previous report found that a diet-induced weight loss of ~ 10 kg in abdominally obese individuals did not improve brachial FMD compared with a control group without weight-loss [40]. In addition, a study of individuals with obesity without diabetes found that weight-loss achieved by either a LCD or a low-fat diet did not affect FMD [19].

There might, however, be transient effects of an increased intake of fat on endothelial function that are attenuated or eventually lost over time. Thus, the brachial FMD increased markedly in response to a low-fat diet for 3 weeks, but not with a VLCD in patients with T2D, even though both groups lost abdominal weight [25]. The authors also found a positive correlation between FMD and protein and fat intake in the low-fat diet group after 3 weeks, but not in the VLCD group. This positive effect of a low-fat diet on FMD was observed despite a significant reduction of HbA1c only in the VLCD group suggesting that a short-term improvement in glycemic control per se does not improve FMD [25]. Intriguingly, an inverted U-shape association between FMD and HbA1c has been reported, with a lower FMD in T2D patients with an HbA1c < 48 mmol/mol than those with an HbA1c of 48–63 mmol/mol and similar to those with an HbA1c higher than that [41]. In contrast, a higher degree of coronary atherosclerosis and lower FMD were observed in T2D patients with a poor glycemic control compared to those with an appropriate glycemic control [42]. In line, a greater improvement in FMD (69%) was reported in T2D patients with poor glycemic control on intensified antidiabetic treatment for 12 months, who achieved an HbA1c ≤ 48 mmol/mol [43]. In our study, the majority (80%) of participants randomized to the LCD achieved an HbA1c ≤ 48 mmol/mol compared with less than half of the participants on the control diet (40%), however, the larger reduction in HbA1c in the LCD group was not accompanied by an improvement in FMD. Furthermore, consistent with other studies [44], we did not observe an association between HbA1c and FMD at baseline. Thus, while we cannot exclude the possibility, that the improvement in HbA1c may have counteracted a potential negative effect of the increased intake of saturated fat on FMD, the lack of associations between FMD and HbA1c at baseline and the lack of changes in other CVD risk factors such as blood lipids and blood pressure suggest that this is not the case.

Markers of systemic low-grade inflammation such as IL-6 and hsCRP are often elevated in obesity and T2D and associated with an increased risk of CVD [26–28]. Moreover, a meta-analysis has shown that weight-loss results a reduction in circulating levels of IL-6 and hsCRP [45], In line, we found that both hsCRP and IL6 levels were positively associated with BMI and abdominal fat (%) at baseline. After 6 months, we found a small but significant reduction in both IL-6 and hsCRP levels in the LCD group, whereas no changes were observed in the control group. At first sight these reductions in IL-6 and hsCRP are in line with a greater weight loss in the LCD group. However, we could not demonstrate a significant difference in change of either IL-6 or hsCRP levels over time between the groups. Thus, this lack of difference in change between the diets was seen despite a greater reduction in body weight and HbA1c in the LCD group. Previous studies reporting the effect of carbohydrate-restriction versus low fat diet for 6 months on pro-inflammatory markers in patients with T2D have shown somewhat variable results [29, 30]. Thus, in one study, plasma IL-6 increased only in response to the low-fat diet, whereas plasma hsCRP remained unchanged in both groups [29]. Conversely, in another study, serum hsCRP decreased only in response to the low-fat diet, whereas serum IL-6 was unchanged in both groups [30]. These changes were observed despite similar weight losses in both diet groups in these studies [29, 30]. However, in line with our findings, none of the studies reported a mean difference in change of IL-6 or hsCRP levels between the groups. Moreover, a randomized cross-over study of a carbohydrate-restricted diet high in protein versus a conventional diet low in fat for 6 weeks in patients with T2D found no changes in either hsCRP or IL-6 levels arguing against a missed short-term effect in our study [46]. Taken together, these results provide evidence that carbohydrate-restriction to different extent (E14% to E34%) does not negatively affect markers of systemic low-grade inflammation. Larger and longer clinical trials are, however, needed to examine if a larger reduction of weight and HbA1c in response to a LCD (E% 10–25%) could reduce systemic low-grade inflammation despite an increased intake of fat.

The strengths of the present study include the randomized design, the well-matched study groups, and the sample size, which according to a non-inferiority analysis was sufficiently large to rule out a change in FMD higher than 20%. In addition, the participants were instructed to maintain their level of physical activity and medication, which allowed us to study the isolated effect of a non-calorie-restricted LCD, which is feasible for patients with T2D under free-living conditions. The limitations include the unblinded (open-label) study design, the lack of strict control with regard to changes in physical activity, medication, and diet macronutrient composition including different types of fat, the latter leading to a higher intake of saturated fat than generally recommended. Moreover, the inability to demonstrate a between-group difference in change of IL-6 and hsCRP levels despite a reduction of both markers in the LCD group suggests that a larger sample size would have been needed to make a conclusion whether a LCD reduces low-grade-inflammation.

In summary, the present study provides evidence that a LCD high in fat for 6 months in patients with T2D instructed to maintain their daily energy intake and level of physical activity does not adversely affect neither the endothelium-dependent (FMD) nor –independent (NID) vasodilation in the brachial artery or selected markers of systemic low-grade inflammation compared with a control diet low in fat. These findings together with the previously reported lack of changes in blood lipids and blood pressure [29] suggest that this nutritional approach does not increase the risk of CVD although studies of longer duration are needed.

### Supplementary Information

**Additional file 1:** **Table S1.** Glycemic control, cardiovascular risk factors, body composition and dietary data.

## Data Availability

The datasets used and/or analyzed during the current study are available from the corresponding author on reasonable request.

## Abbreviations

DPP4: Dipeptidyl peptidase-4
DXA: Dual-energy X-ray absorptiometry
E%: Energy per cent
GLP-1: Glucagon-like peptide-1
LCD: Low-carbohydrate diet
MUFA: Monounsaturated fatty acids
PUFA: Polyunsaturated fatty acids
SGLT2: Sodium-glucose cotransporter-2
SFA: Saturated fatty acids
VLCKD: Very low-carbohydrate ketogenic diet
