# Supplementary material for: A six-month low-carbohydrate diet high in fat does not adversely affect endothelial function or markers of low-grade inflammation in patients with type 2 diabetes: an open-label randomized controlled trial
Source: Cardiovasc Diabetol. 2023 Aug 17;22:212. doi: 10.1186/s12933-023-01956-8 (PMC10436534; doi:10.1186/s12933-023-01956-8)
Supplement: Supplementary file 1 — Additional file 1: Table S1. Glycemic control, cardiovascular risk factors, body composition and dietary data. [file 12933_2023_1956_MOESM1_ESM.docx]

**Table S1. Glycemic control, cardiovascular risk factors, body composition and dietary data**

|  | **Baseline** | | **6 months** | |  | |
| --- | --- | --- | --- | --- | --- | --- |
|  | **LCD**  **(n = 49)** | **Control**  **(n = 21)** | **LCD**  **(n = 44)** | **Control**  **(n = 20)** | **MDIC** | ***p*-value** |
| HbA1c (mmol/mol) | 54.3 ± 9.6 | 56.5 ± 6.6 | 43.5 ± 6.5 | 53.2 ± 9.5 | -7.4 ± 2.1 | < 0.0001 |
| Systolic BP (mmHg) | 134 ± 14 | 141 ± 13 | 131 ± 13 | 136 ± 11 | 0.2 ± 2.9 | 0.936 |
| Diastolic BP (mmHg) | 85 ± 9 | 85 ± 9 | 84 ± 7.7 | 84 ± 8 | 1.2 ± 1.9 | 0.538 |
| Serum LDL (mmol/l) | 2.3 ± 0.8 | 2.4 ± 0.8 | 2.4 ± 0.8 | 2.2 ± 0.9 | 0.3 ± 0.2 | 0.078 |
| Serum HDL (mmol/l) | 1.2 ± 0.2 | 1.1 ± 0.3 | 1.3 ± 0.3 | 1.1 ± 0.3 | 0.1 ± 0.0 | 0.128 |
| Serum TG (mmol/l) | 1.91 ± 1.2 | 2.14 ± 1.2 | 1.42 ± 0.7 | 1.66 ± 0.7 | -0.02 ± 0.22 | 0.920 |
| BMI (kg/m^2^) | 32.5 ± 6.0 | 35.5 ± 6.8 | 30.7 ± 6.1 | 34.9 ± 6.8 | -1.3 ± 0.5 | 0.004 |
| Weight (kg) | 97.8 ± 22.1 | 103.1 ± 20.3 | 92.6 ± 23.5 | 101.9 ± 19.5 | -3.8 ± 1.3 | 0.004 |
| Waist circumference (cm) | 110 ± 15 | 116 ± 14 | 103 ± 15.4 | 114 ± 14.9 | -5 ± 1 | < 0.001 |
| Hip circumference (cm) | 108 ± 12 | 114 ± 14 | 105 ± 12 | 112 ± 14 | -2 ± 1 | 0.105 |
| Serum creatinine (µmol/l) | 71.3 ± 15.4 | 73.2 ± 21 | 68.2 ± 14.8 | 72.7 ± 22.0 | -1.3 ± 2.2 | 0.549 |
| **DXA scans** | **(n = 49)** | **(n = 21)** | **(n = 44)** | **(n = 20)** |  |  |
| Total lean mass (kg) | 56.9 ± 13.1 | 57.9 ± 9.7 | 54.8 ± 14.2 | 57.9 ± 9.9 | -1.3 ± 0.6 | 0.018 |
| Total fat mass (kg) | 38.6 ± 12.8 | 42.7 ± 13.6 | 35.7 ± 13.2 | 41.3 ± 13.0 | -2.2 ± 1.0 | 0.029 |
| Total lean mass (%) | 58.3 ± 7.6 | 56.8 ± 7.1 | 59.1 ± 7.9 | 57.4 ± 7.4 | 1.0 ± 0.6 | 0.071 |
| Total body fat (%) | 38.9 ± 7.9 | 40.7 ± 7.5 | 37.9 ± 8.3 | 40.0 ± 7.7 | -1.2 ± 0.6 | 0.043 |
| **Dietary data** | **(n = 36)** | **(n = 18)** | **(n = 39)** | **(n = 19)** |  |  |
| Kcal per day | 1805 ± 464 | 1817 ± 425 | 1642 ± 387 | 1660 ± 517 | -24 ± 121 | 0.841 |
| Carbohydrates (E%) | 42.1 ± 7.4 | 47.0 ± 5.7 | 13.4 ± 7.5 | 48.4 ± 4.3 | -30.0 ± 2.7 | < 0.001 |
| Protein (E%) | 19.4 ± 4.9 | 19.0 ± 3.4 | 23.4 ± 4.5 | 22.5 ± 4.4 | 0.5 ± 1.7 | 0.773 |
| Total fat (E%) | 38.2 ± 7.6 | 33.3 ± 6.7 | 63.2 ± 7.8 | 28.3 ± 4.5 | 29.9 ± 2.7 | < 0.001 |
| SFA (g/day) | 24.8 ± 10.0 | 20.5 ± 12.0 | 40.7 ± 13.8 | 15.6 ± 5.7 | 20.5 ± 3.7 | < 0.001 |
| MUFA (g/day) | 20.8 ± 9.8 | 16.4 ± 7.1 | 33.0 ± 11.3 | 12.5 ± 4.8 | 16.0 ± 3.3 | < 0.001 |
| PUFA (g/day) | 8.6 ± 4.4 | 7.6 ± 1.9 | 15.8 ± 7.7 | 7.0 ± 2.9 | 7.6 ± 1.6 | < 0.001 |
| SFA (E%) | 12.3 ± 3.7 | 9.8 ± 4.2 | 22.4 ± 5.8 | 8.5 ± 1.7 | 11.3 ± 1.4 | < 0.001 |
| MUFA (E%) | 10.3 ± 3.9 | 8.1 ± 2.7 | 17.9 ± 3.8 | 6.8 ± 1.7 | 8.9 ± 1.4 | < 0.001 |
| PUFA (E%) | 4.2 ± 1.6 | 3.9 ± 0.9 | 8.5 ± 3.0 | 3.8 ± 1.0 | 4.4 ± 0.7 | < 0.001 |

MDICs are reported as β-coefficient ± SE. Other data are given as means ± SD. Abbreviations: Mean difference in change, MDIC; BP, blood pressure; DXA, dual-energy x-ray absorptiometry; E%, percent of total energy intake; TG, triglycerides; SFA, saturated fatty acids; MUFA, monounsaturated fatty acids; PUFA, polyunsaturated fatty acids.
